# Disseminated cryptococcal infection with pulmonary involvement presenting as diffuse cavitary nodules in an immunocompromised patient: a case report

**DOI:** 10.1186/s12890-023-02332-8

**Published:** 2023-01-27

**Authors:** Jinbao Huang, Hongyan Li, Changqing Lan, Heng Weng

**Affiliations:** 1 Department of Respiratory Medicine, People’s Hospital, Affiliated to Fujian University of Traditional Chinese Medicine, Fuzhou, 350004 China; 2 Department of Critical Care Medicine, People’s Hospital, Affiliated to Fujian University of Traditional Chinese Medicine, Fuzhou, 350004 China; 3 Department of Radiology, Fuzhou Pulmonary Hospital of Fujian, Educational Hospital of Fujian Medical University, Fuzhou, 350008 China

**Keywords:** Cryptococcosis, Meningitis, Amphotericin B liposome, X-ray computed tomography

## Abstract

**Background:**

Disseminated cryptococcal infection is especially prone to occur in immunosuppressed hosts. We herein report the case of an immunosuppressed girl with disseminated cryptococcal infection in whom pulmonary cryptococcosis (PC) presented as diffuse cavitary pulmonary nodules, a finding which has rarely been reported.

**Case presentation:**

A 16-year-old immunocompromised girl presented with fever and a non-productive cough. A chest computed tomography (CT) scan revealed diffuse pulmonary nodules with cavities. Subsequent results were consistent with disseminated cryptococcosis with *Cryptococcus* identified in her blood, bone marrow and cerebrospinal fluid cultures. Thus, the patient was diagnosed with disseminated cryptococcal infection with PC, cryptococcus meningitis, cryptococcus osteomyelitis and cryptococcus sepsis. After antifungal treatment, the patient demonstrated both clinical and chest radiological improvement.

**Conclusion:**

The atypical clinical manifestations of a disseminated cryptococcal infection and the rare manner of chest CT findings of PC reported in our case are easy to misdiagnose. It is necessary to carry out a thorough search for a definitive diagnosis using various methods.

## Background

Cryptococcosis is an invasive fungal infection caused by a ubiquitous organism that has a pulmonary portal of entry [1]. It is more common in immunocompromised hosts; hence, one of the principal predisposing factors is human immunodeficiency virus (HIV) infection [2]. Other factors which increase the risk of infection are chemotherapy and radiotherapy treatment for malignant tumours, long-term administration of immunosuppressants or corticosteroids, severe diabetes mellitus, liver cirrhosis, chronic kidney disease with dialysis therapy, and other immunodeficiency diseases [3, 4]. Disseminated cryptococcal infection is especially prone to occur in immunosuppressed hosts. We herein reported the case of an immunosuppressed girl with disseminated cryptococcal infection in whom pulmonary cryptococcosis (PC) presented as diffuse cavitary pulmonary nodules, a finding which has rarely been reported.

## Case presentation

**Fig. 1:**
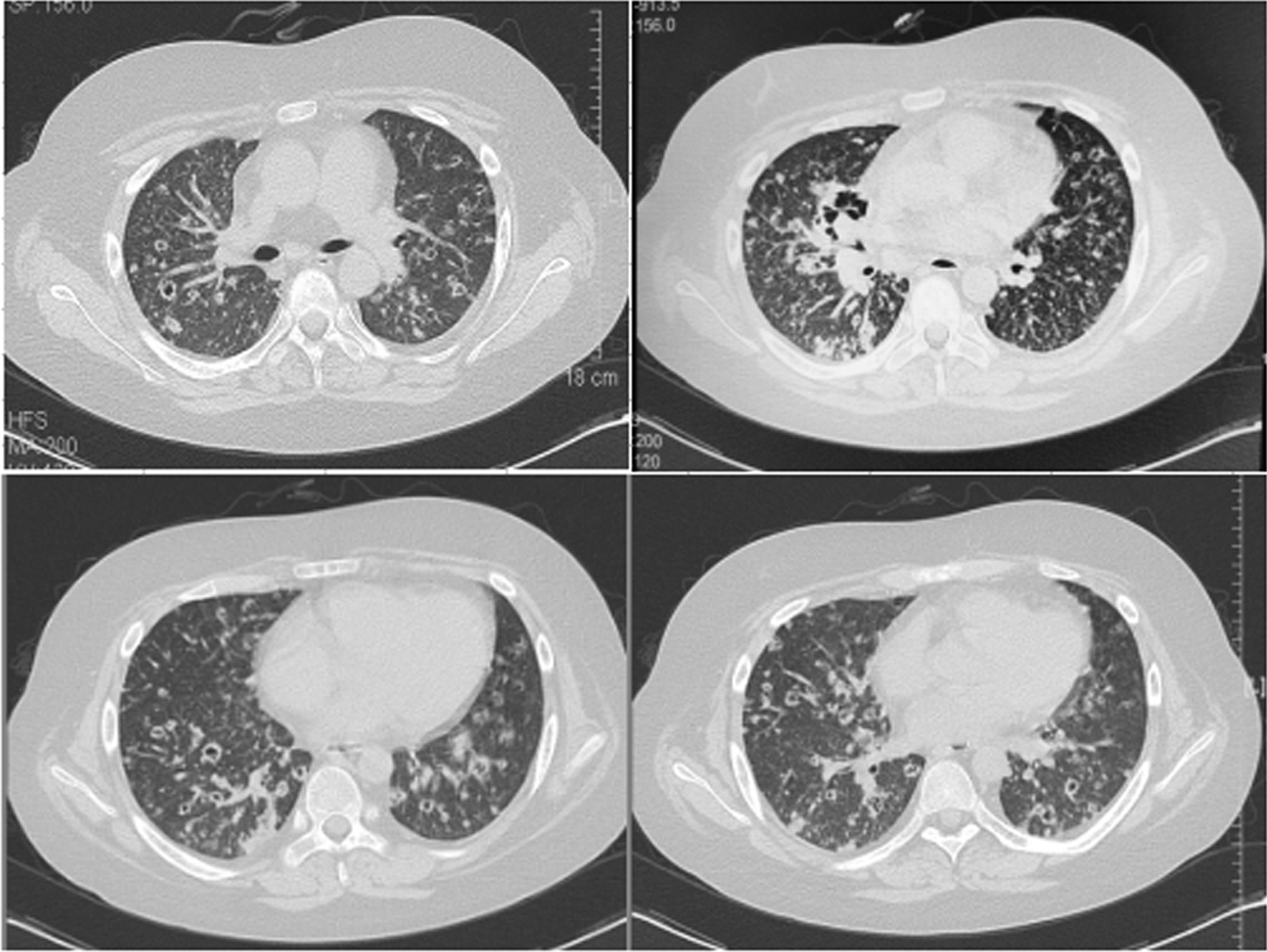
Chest computed tomography demonstrating a thick-walled cavitating lesion 2.5 cm × 3.5 cm in size in the right middle lobe with diffuse small nodules and cavities in both lungs

A 16-year-old girl was admitted to our hospital complaining of a dry cough, fever, chills and fatigue for a period of 10 days. No other symptoms such as headaches, nausea, vomiting, or night sweats were noted. The patient was 165 cm in height and 67 kg in weight. She had a history of yolk sac tumour for which she had undergone uterus and bilateral adnexectomy and post-surgery antineoplastic chemotherapy. Further, she had a history of idiopathic thrombocytopenic purpura which was treated with long-term corticosteroid therapy. She had received cefoperazone sulbactam sodium for presumed pneumonia for a week in a local community hospital prior to admission but failed to improve. Physical examination revealed obesity, moon face, and multiple dark red bruises on her entire skin. Auscultation of her chest revealed decreased breath sounds in both lungs but no dry or wet rales. Signs of meningeal irritation and pathological reflexes were negative. Haematologic and biochemical analyses were unremarkable, except for elevated hypersensitive C-reactive protein levels and a white blood cell count of 13.7 × 10^9^/L (normal, 4.0–10.0 × 10^9^/L). Dynamic monitoring of peripheral blood showed that lymphocytes gradually decreased from 1.0 × 10^9^/L to 0.7 × 10^9^/L (normal, 0.8–4.0 × 10^9^/L). Serum tumour markers were also unremarkable and the HIV-antibody test was negative. Arterial blood gas pressures were: power of hydrogen, 7.466; pressure of oxygen, 70.1 mmHg (9.3 kPa); pressure of carbon dioxide, 35.3 mmHg (4.7 kPa); and arterial oxygen saturation, 95.0%. A chest computed tomography (CT) scan showed diffuse military pattern of pulmonary micronodules and small nodules with multiple cavities of different sizes throughout the lungs as well as a focal thick-wall cavitating lesion 2.5 × 3.5 cm in size on the right hilum (Fig. 1). Small lymphadenopathies which were < 1.5 cm in diameter were observed in the mediastinum. Abdominal CT and abdominal ultrasound scans both revealed fatty liver and left kidney stones. An enhanced brain CT scan did not identify any abnormalities.

**Fig. 2:**
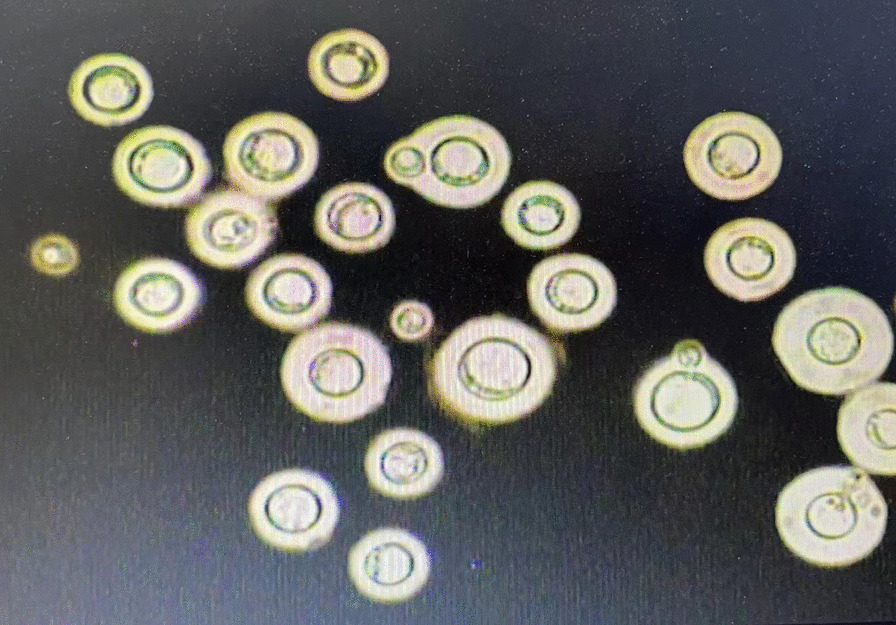
India ink examination of a CSF specimen demonstrating the typical transparent thick capsule of cryptococcus spores. India ink stain was shown as 400 ×. Microscope type: OLYMPUS CX41; acquisition software: RCZ 600 Multifunctional Microscopic Image Processin System

**Fig. 3:**
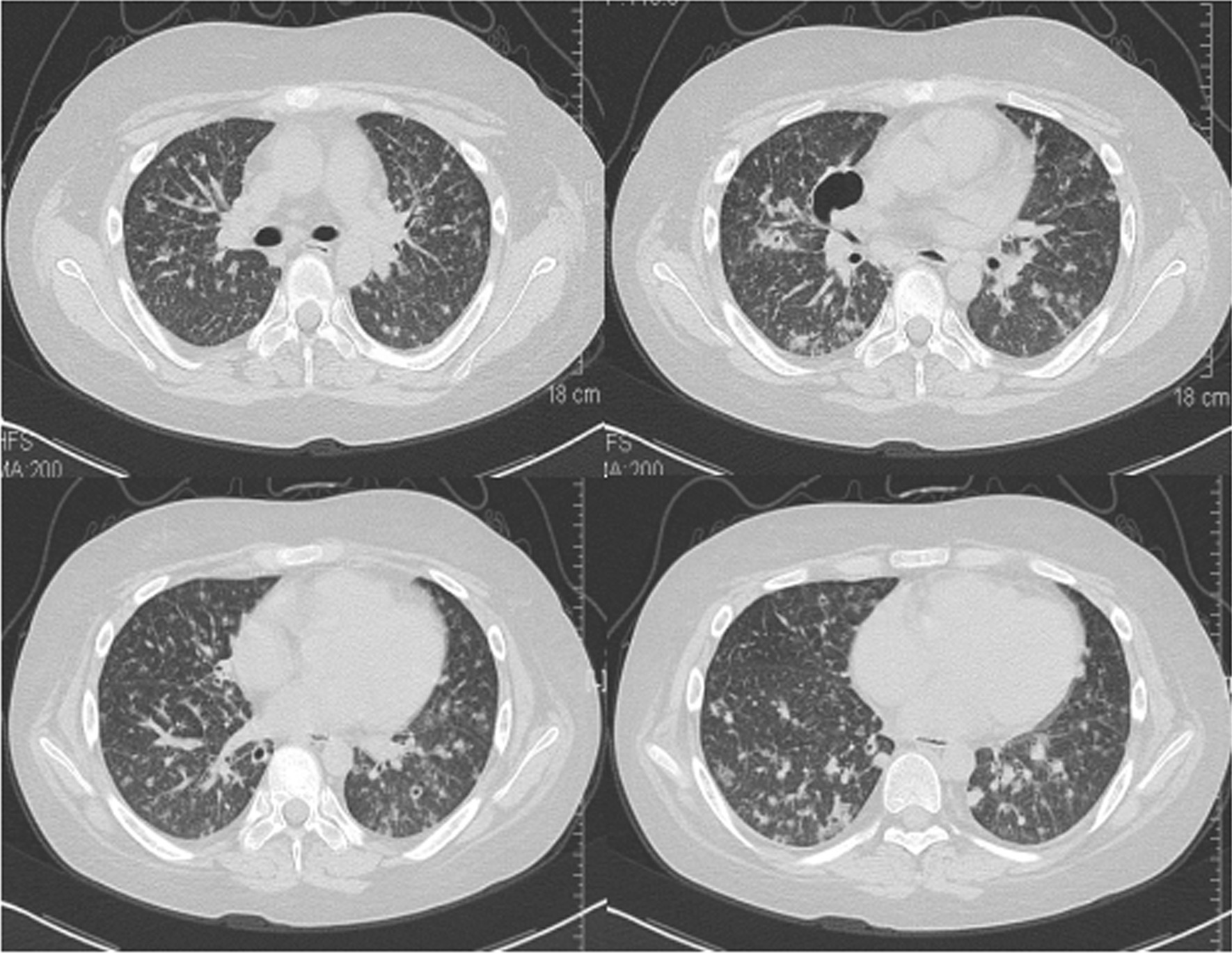
After a 10-week treatment, repeated chest computed tomography demonstrateing significant lesion absorption

The primary diagnosis was indeterminate pulmonary nodules which could be malignant, such as primary pulmonary cancer or metastatic tumour, or benign lesions, such as disseminated pulmonary tuberculosis or a bacterial lung abscess. A bronchoscopy was then performed, but bronchoalveolar lavage fluid (BALF) was negative for bacteria, fungi, and mycobacteria. Results of a transbronchial lung biopsy (TBLB) were also unremarkable. We administered empirical treatment for haematogenous lung abscess with linezolid. However, the fever persisted and mainly manifested as an afternoon fever. Due to the clinical possibility of haematogenous disseminated pulmonary tuberculosis which is prone to be complicated by tuberculous meningitis, a lumbar puncture was later performed. The analysis of cerebrospinal fluid (CSF) showed an intracranial pressure of 160 mm H_2_O (normal, 70–180 mm H_2_O), protein of 458.3 mg/L, glucose of 3.1 mmol/L, and chloride of 115.4 mmol/L. CSF detection was negative for bacteria and acid-fast bacilli, whereas CSF stained with India ink was positive for *C. neoformans* (Fig. 2). Further results of blood, bone marrow and CSF cultures by sabouraud agar plate medium were also positive for *C. neoformans*. The patient was diagnosed with disseminated cryptococcal infection involving both lungs, the bone marrow, bloodstream and cerebral tissue and the aforementioned medication was discontinued. To reduce the adverse drug reactions (renal function, gastrointestinal reaction, etc.), the patient was initially treated with intravenous fluconazole (Diflucan) 400 mg qd (800 mg of starting dose) and 5-fluorocytosine 150 mg qid (100 mg·kg^−1^·d^−1^). After one week treatment, her temperature and fever duration gradually decreased. However, repeated CSF testing and blood culture were persistently positive for *C. neoformans*. Signs of meningeal irritation and pathological reflexes were also noted at that time. Repeated chest CT scan revealed cavitary lesions enlarged in both lungs. Due to the high bacterial load and wide dissemination, an dditional treatment with amphotericin B liposome (AmBisome) 60 mg qd (1 mg·kg^−1^·d^−1^) was adminitrated, and the fever eventually disappeared after 3 days. Although the highest recommended dose of AmBisome is 3 mg·kg^−1^·d^− 1^, the patient could not tolerate a higher dose of AmBisome due to several adverse drug effects including repeated severe hypokalaemia, palpitation, high blood pressure and gastrointestinal reactions. Despite this, after a 10-week treatment, the patient’s symptoms disappeared and a follow-up CT scan revealed significant lesion absorption in both lungs as well as a marked improvement of the cavitating right middle lobe lesion (Fig. 3). Due to stabilization of her condition, the patient was transferred to the local community hospital for continued treatment. Five months after the hospital discharge, the relevant follow-up patient information was obtained by telephone. Data showed *C. neoformans* negative CSF specimens, and remarkable absorption of cryptococcal lesions and closure of cavities on the chest CT image after subsequent treatment with fluconazole.

## Discussion

Disseminated cryptococcosis tends to occur in immunosuppressed patients and, due to infection via inhalation of cryptococcus aerosol, the lungs are often involved [5]. In the case presented here, the patient was immunosuppressed due to yolk sac tumour-related treatments as well as long-term corticosteroid treatment for idiopathic thrombocytopenic purpura. Lymphopenia was also noted, indicating a decline in immunity. The patient only presented with dry cough, high fever, chills and fatigue after onset, and symptoms such as headache, nausea, vomiting, meningeal irritation and pathological signs were not noted, which might be attributed to being in the early course of the disease and the absence of intracranial hypertension (only 160 mm H_2_O) upon admission. Chest CT scan showed a hilar mass in the right lung with diffuse pulmonary nodules with cavities in both the lungs. Given the patient’s history of cancer and immunosuppression, lung metastasis and pulmonary infectious diseases (pulmonary tuberculosis and lung abscess) were considered upon admission; thus, a bronchoscopy was performed. However, bacteria inspection of BALF was negative and pathological examinations of TBLB were also unremarkable. Serum tumour markers were also negative, and empirical treatment with anti-bacterium drugs was non-effective. All the above results did not support bacterial lung abscesses or lung malignancies. Even so, since the patient presented with a dry cough and an afternoon fever, combined with diffuse nodular lesions in both lungs, hematogenous disseminated pulmonary tuberculosis could not be excluded. Since this type of tuberculosis is prone to be complicated by tuberculous meningitis, CSF testing was carried out and the result was positive for *C.* neoformans. This provided a definite diagnosis of cryptococcal meningitis. In addition, due to the low detection rate of* Cryptococcus* in lower respiratory tract specimens (sputum and BALF) [6], the diagnosis of pulmonary cryptococcosis in most cases is still mainly dependent on the pathological biopsy of the lung tissue [6, 7]. Unfortunately, no positive results of TBLB were found in this patient, and the diagnosis of PC could not be obtained timely. Although the bronchoscopy did not assist in the diagnosis of PC, it was still helpful in differentiating pulmonary tuberculosis, lung abscesses, and lung tumours for our patient.

The chest imaging of PC is diverse, and includes nodule and masses, consolidation, pleural effusion, etc., with the most common radiological finding being single or multiple nodules [8–12]. PC can present as an intrapulmonary cavity, with an incidence of 11.0–34.6% [11–13], but bilateral diffuse cavity nodules are rare. In this case, the chest CT image showed randomly distributed diffuse nodules, which was different from the common imaging characteristics of cryptococcal intra-airway dissemination, and were consistent with blood-borne transmission in the lung. Further, the patient had a history of malignant tumours and underlying immunosuppressive diseases, which could easily be confused with other diseases such as hematogenous disseminated pulmonary tuberculosis, pulmonary metastasis, and blood-borne lung abscess. Although pulmonary involvement in disseminated cryptococcosis is typically caused by inhalation of cryptococcus aerosol, the lungs can be re-infected by cryptococci carried around in the bloodstream. In this case, cryptococcal infection involved the bloodstream, and PC was considered to be caused by blood-borne transmission of *Cryptococcus* in the lung.

Both the clinical manifestations and chest imaging characteristics in this case lacked specificity; thus, it was difficult to achieve a definite diagnosis. To look for further diagnostic clues, an additional of bronchoscopy, blood culture, bone marrow, and CSF examination were performed. The patient tested positive for *C. neoformans* on CSF stain and culture, as well as blood and bone marrow cultures. Following a 10-week antifungal treatment, both clinical and chest radiological improvement were observed. Thus, the patient was diagnosed with disseminated cryptococcal infection with PC, cryptococcus meningitis, cryptococcus osteomyelitis and cryptococcus sepsis which was cured with anti-fungal treatment.

In conclusion, the atypical clinical manifestations of disseminated cryptococcal infection and the rare presentation of chest CT findings of PC reported in our case would have made it prone to misdiagnose. Indeed, it was difficult to obtain a diagnosis based on only the limited clinical data. Therefore, in such cases it is worth pursuing a thorough search for a definitive diagnosis via various diagnostic methods.

## Data Availability

All data generated or analysed during this study are included in this published article.

## Abbreviations

AmBisome: Amphotericin B liposome
BALF: Bronchoalveolar lavage fluid
CSF: Cerebrospinal fluid
CT: Computed tomography
HIV: Human immunodeficiency virus
ITP: Idiopathic thrombocytopenic purpura
PC: Pulmonary cryptococcosis
TBLB: Transbronchial lung biopsy
